# Effects of Biochar–Nitrogen Interaction on Soil Nitrogen Transformation and Cucumber Growth in Facility Cultivation

**DOI:** 10.3390/plants15111658

**Published:** 2026-05-28

**Authors:** Bing Bai, Xue Yang, Qing An, Xia Cao, Ning Zhang, Mingjia Tang, Chuncheng Wu, Yingbin Qi

**Affiliations:** 1 College of Horticultural Science & Technology, Hebei Normal University of Science & Technology, Qinhuangdao 066004, China; baibing1112@126.com (B.B.); yangxue19990122@163.com (X.Y.); m18840551887@163.com (Q.A.); caoxia_xy@163.com (X.C.); zhangning9097@163.com (N.Z.); 2 Hebei Key Laboratory of Horticultural Germplasm Excavation and Innovative Utilization, Qinhuangdao 066004, China; 3 Hebei Higher Institute Application Technology Research and Development Center of Horticultural Plant Biological Breeding, Qinhuangdao 066004, China; 4 Dafang County Bureau of Agriculture and Rural Affairs, Bijie 551600, China; 5 National Key Laboratory of Crop Genetics and Germplasm Enhancement, College of Horticulture, Nanjin Agricultural University, Nanjing 210095, China; t2024074@njau.edu.cn

**Keywords:** facility continuous cropping soil, biochar, nitrogen cycling, soil microorganisms

## Abstract

Continuous cropping and long-term excessively using nitrogen fertilizers in facilities vegetables has led to the imbalance of nitrogen conversion in soil and plants. A pot experiment was conducted to investigate the effects of biochar on soil and plant nitrogen transformation in cucumber under simulated different nitrogen contents in facility cultivation. Eight pot treatments: no nitrogen (N0), 100 kg·hm^−2^ of nitrogen (N100), 150 kg·hm^−2^ of nitrogen (N150), 200 kg·hm^−2^ of nitrogen (N200), and which with the addition of 5% biochar named BN0, BN100, BN150, BN200. The results showed that BN100 (100 kg·hm^−2^ N + 5% biochar) significantly increased the soil nitrogen transformation and the growth of cucumber. Specifically, the nitrate reductase, GOGAT activity, and the nitrate nitrogen absorption of cucumber roots were enhanced. It also elevated soil pH by 0.43 units and increased urease, neutral protease, nitrite reductase (NIR, and NR activities by 136.63%, 23.95%, 18.4%, and 12.1%, respectively. The relative abundance of nitrogen metabolism-related microorganisms such as *Nitrospira* and *Sphingomonas* were increased. The nitrification, nitrogen fixation, the contents of nitrate nitrogen and nitrite nitrogen in soil were increased. These changes collectively improved soil nitrogen transformation and ultimately promoted cucumber plant growth. This study reveals the potential role of biochar in regulating soil nitrogen transformation and facilitating plant growth.

## 1. Introduction

Nitrogen (N) is one of the essential macronutrients for plant growth, and rational application of nitrogen fertilizer is crucial for vegetable production. Cucumber (*Cucumis sativus* L.) is one of the most widely cultivated vegetable crops in protected agriculture worldwide. Cucumber is highly sensitive to N supply: insufficient N limits vegetative growth and yield, while excessive N causes vigorous vegetative growth. In facility cucumber production, continuous cropping is common due to the pursuit of economic benefits, coupled with excessive application of nitrogen fertilizer, leading to nitrogen accumulation in soil [1,2,3,4]. Excess nitrogen of the soil resulted in a decrease in nitrogen use efficiency, causes nitrogen loss and deterioration of the soil environment. In addition, it leads to a decline in soil pH and organic matter content [5], further exacerbating secondary salinization of the soil [6], ultimately causing the vegetables growth obstacles [4].

Excessive nitrogen fertilizer application reduces soil enzyme activities. Soil enzyme play positive roles in promoting soil substance transformation [7,8,9,10], and their activities can reflect soil quality levels [11]. Liu et al. found that long-term overapplication of chemical fertilizer would reduce the activities of soil sucrase, urease and alkaline phosphatase [12]. Du et al. found that continuous application of nitrogen fertilizer would reduce the relative abundance of microorganisms involved in nitrogen metabolism in soil [13]. Li et al. showed that the application of nitrogen fertilizer affected the abundance of genes related to soil denitrification, assimilation and nitrate reduction [14]. Biochar application improved soil pH, promoted the formation of organic matter, and reduced the loss of soil nutrients, due to the unique structure and adsorption capacity [14]. Biochar addition also enhances soil nitrogen transformation [15,16,17]. Zheng et al. [18] found that biochar significantly improved soil ammonia-oxidizing microbial communities, affected soil nitrification processes, and increased soil net nitrification rates. Lin et al. [19] found that biochar application increased biological nitrogen fixation in soybean by improving soil physical and chemical properties. Biochar can adsorb and remove soil nitrification inhibitors, thus regulating soil N transformation by alleviating nitrification inhibition [13]. The application of biochar could promote the growth of cucumber seedlings, optimize root architecture, affect the soil nitrogen absorption and transformation by the roots [20]. Biochar also promotes the growth of nitrogen-fixing bacteria and enhances soil nitrogen cycling and transformation [21].

Although previous studies have confirmed that biochar can regulate soil N transformation and crop growth, the interactive mechanisms among soil N cycling, root N metabolism, and rhizosphere microbial communities under excessive N accumulation in facility continuous cropping soils remain unclear. We hypothesized that biochar amendment would improve soil N transformation, enhance root N absorption and assimilation, and optimize rhizosphere microbial communities, thereby promoting cucumber growth under different N supply levels.

In this study, a pot experiment was conducted to simulate different nitrogen content levels in facility cultivation, and the effects of biochar on soil nitrogen transformation, root nitrogen metabolism, and rhizosphere microbial community of cucumber were systematically studied. The results can provide a theoretical basis and practical reference for rational nitrogen fertilizer application and efficient biochar utilization in facility cucumber production.

## 2. Results and Analysis

### 2.1. Cucumber Plant Growth

Forty-five days after cucumber transplanting, plant height and chlorophyll content first increased then decreased with rising N input (Table S1). BN0 and BN100 increased plant height by 21.04% and 15.78% relative to N0 and N100, respectively. BN100 had the highest chlorophyll content, 108.67% higher than N100. Biochar had no significant effect on stem diameter or shoot dry weight.

### 2.2. Nitrogen Content in Cucumber Plants and Root Metabolism Enzymes

**Figure 1:**
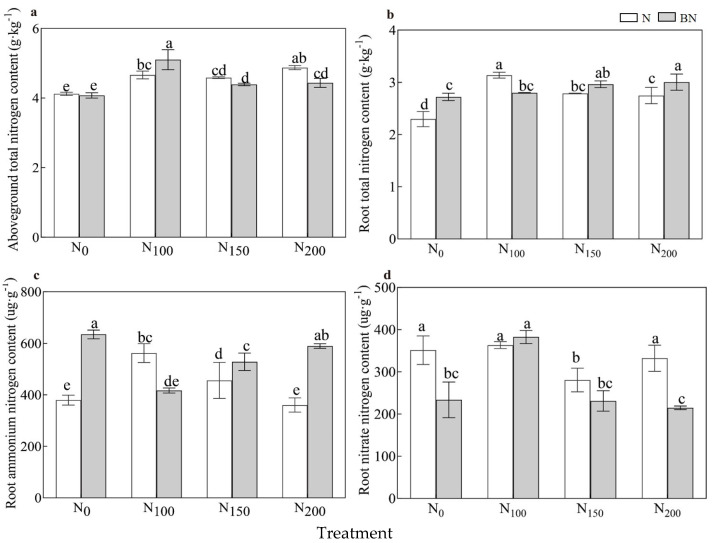
Effects of biochar–nitrogen interaction on nitrogen contents in cucumber plants. Note: (**a**): Aboveground total nitrogen content; (**b**): Root total nitrogen content; (**c**): Root ammonium nitrogen content (**d**): Root nitrate nitrogen content. Different lowercase letters indicate significant differences between treatments (*p* < 0.05).

**Figure 2:**
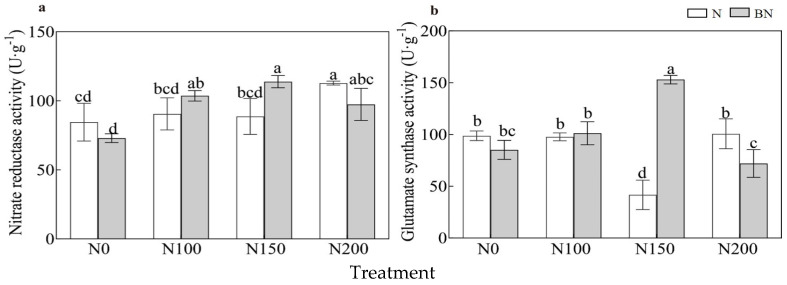
Effects of biochar–nitrogen interaction on root nitrogen metabolism enzyme activities. Note: (**a**): Nitrate reductase activity; (**b**): Glutamate synthase enzyme activity. Different lowercase letters indicate significant differences between treatments (*p* < 0.05).

With the increase nitrogen content of soil, the contents of total nitrogen, ammonium nitrogen, and nitrate nitrogen in the roots without biochar addition generally exhibited a trend of first increasing and then decreasing (Figure 1). The nitrate nitrogen content of BN100 treatment was the highest with 5.27% higher than that in the N100 treatment. Compared with the treatment without biochar addition, both BN100 and BN150 treatments increased the activity of nitrate reductase (NR) in the roots. Similarly, BN100 and BN150 treatments also enhanced the glutamate synthase (GOGAT) activity in the roots, whereas the GOGAT activity in the cucumber roots of other biochar treatments was lower than that in the treatment without biochar addition (Figure 2). It can be concluded that biochar addition enhanced the activities of nitrogen metabolism enzymes in cucumber roots, improved the nitrate nitrogen assimilation capacity of the roots, and thus contributed to promoting the nitrate nitrogen uptake by plants.

### 2.3. Soil Chemical Properties, Contents of Different Forms of Nitrogen, and Soil Nitrogen Metabolism Enzyme

As can be seen from Table 1, with the increase in nitrogen content, the soil pH value showed an overall decreasing trend, while the soil pH value of the BN100 treatment was significantly higher than that of other treatments. The content of soil alkali-hydrolyzable nitrogen increased with the rise in soil nitrogen content, but the soil alkali-hydrolyzable nitrogen content in biochar-amended treatments was lower than that in the treatment without biochar addition. Both the soil available K and organic matter contents in biochar-amended treatments were higher than those in the treatment without biochar addition. As for the soil available P content, except for the BN200 treatment, the soil available P contents in other biochar-amended treatments were higher than that in the treatment without biochar addition, but the differences were not significant.

As shown in Table 2, with the increase in nitrogen content, the contents of soil total nitrogen (TN), nitrate nitrogen (NO_3_^−^-N) and nitrite nitrogen (NO_2_^−^-N) all showed a trend of first increasing and then decreasing. However, biochar addition increased the contents of soil TN and NO_3_^−^-N in all treatments. It can be concluded that under soil conditions with different nitrogen contents, biochar addition could increase the contents of different forms of nitrogen in soil, among which the BN100 treatment had the most significant promoting effect. This treatment was conducive to facilitating the conversion of ammonium nitrogen to nitrite nitrogen and nitrate nitrogen in soil, thereby enhancing the nitrogen uptake capacity of plants.

**Figure 3:**
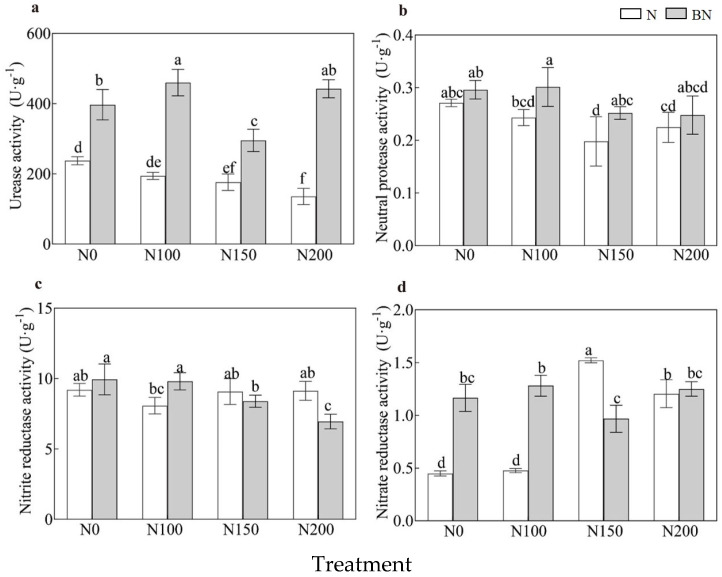
Effects of biochar–nitrogen interaction on the enzyme activities of soil nitrogen metabolism. Note: (**a**): Urease enzyme activity; (**b**): Neutral protease enzyme activity; (**c**): Nitrate reductase enzyme activity; (**d**): Nitrite reductase enzyme activity. Different lowercase letters indicate significant differences between treatments (*p* < 0.05).

It can be seen from Figure 3 that with the increase in soil nitrogen content, the soil urease activity (UE) and neutral protease activity (NPA) in the treatment without biochar addition both showed a decreasing trend. Biochar addition significantly increased the soil urease activity in all corresponding treatments; the soil UE activity of the BN100 treatment was the highest, which was significantly 136.63% higher than that of the N100 treatment. The soil NPA of the BN100 and BN150 treatments was significantly 23.95% and 27.29% higher than that of the N100 and N150 treatments, respectively, while there were no significant differences among other treatments. The soil nitrate reductase (NR) activity showed a trend of first increasing and then decreasing with the increase in nitrogen content. Except for the BN150 treatment, the soil NR activity in other biochar-amended treatments was higher than that in the treatment without biochar addition.

### 2.4. Soil Microbiomes

After quality filtering, 1,313,111 sequences were clustered with 546,288,130 bp. An average sequence length of 416 bp, a minimum length of 203 bp, and a maximum length of 528 bp (Table S2). The coverage rate of the bacterial communities in each treatment was higher than 0.97 (Figure S1). Which indicated that the sequencing depth met the requirements, and the results could truly reflect the conditions of various samples. Alpha diversity analysis showed that both N0 and N100 treatments reduced the bacterial Shannon and Chao indices, while BN0 and BN100 significantly increased the bacterial Shannon and Chao indices (Figure S1). Which indicated that an increase in soil nitrogen content reduced abundance and diversity of bacterial community, whereas the addition of biochar treatment enhances them.

**Figure 4:**
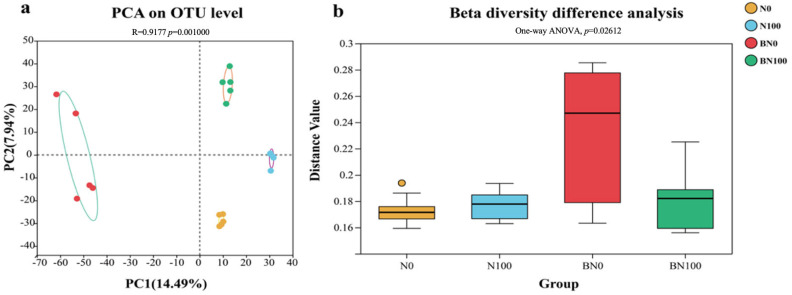
Effects of biochar–nitrogen interaction on the beta diversity scores of soil bacterial communities. (**a**): PCA on OUT level; (**b**): Bate diversity.

PCA analysis level revealed a distinct separation between biochar-amended and non-amended soils (PC1 was 14.49%, PC2 was 7.94%) with the OTU. N0, N100, and BN100 clustered closely together, indicating minimal differences in their bacterial community compositions. In contrast, BN0 was distinctly distant from the other three treatments, suggesting a unique bacterial community structure. These findings demonstrated that biochar application had a significant impact on the bacterial community structure of the cucumber rhizosphere soil (Figure 4a). And Beta diversity analysis corroborated these results, confirming that biochar amendment significantly altered the bacterial community structure across soils with different nitrogen levels (Figure 4b).

**Figure 5:**
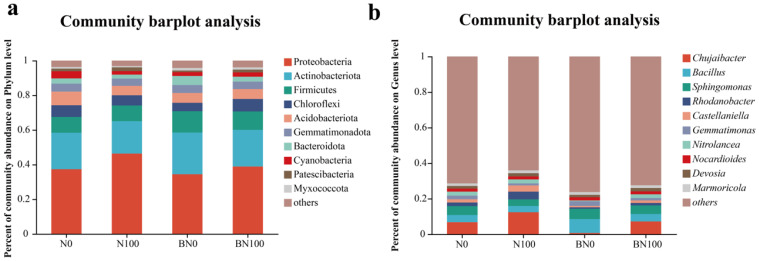
Effects of biochar–nitrogen interaction on the composition of soil microbial community. (**a**) Bacterial phylum level community composition. (**b**) Bacterial genus level community composition.

At the phylum level (Figure 5a), the dominant groups of all samples were *Proteobacteria*, *Actinobacteriota*, *Firmicutes*, *Chloroflexi*, *Acidobacteriota*, *Gemmatimonadota*, *Bacteroidota*, *Cyanobacteria*, *Patescibacteria*, and *Myxococcota*. The relative abundances of *Actinobacteriota*, *Firmicutes*, and *Chloroflexi* in treatment BN100 were higher than those in N100. At the genus level (Figure 5b), the dominant groups across all samples were *Chujaibacter*, *Bacillus*, *Sphingomonas*, *Rhodanobacter*, *Castellaniella*, *Gemmatimonas*, *Nitrolancea*, *Nocardioides*, *Devosia*, and *Marmoricola*. Compared with treatment N100, BN100 increased the relative abundances of *Bacillus*, *Sphingomonas*, *Gemmatimonas*, and *Devosia*.

**Figure 6:**
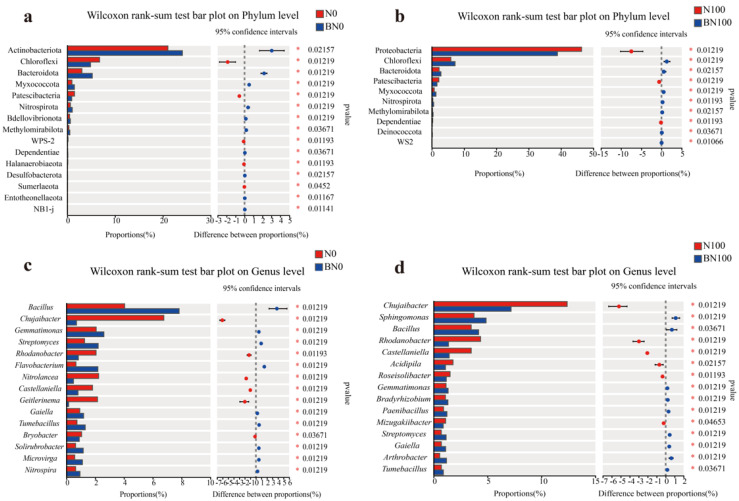
Effects of biochar–nitrogen interaction on the differential flora of soil bacterial microorganisms at phylum and genus level. Note: (**a**) Phylum-level differential flora of N0, BN0; (**b**) Phylum-level differential flora of N100, BN100; (**c**) Genus-level differential flora of N0, BN0; (**d**) Genus-level differential flora of N100, BN100.

To further clarify the differences in microbial composition among the various treatments, the Wilcoxon rank-sum test was employed to analyze the effects of biochar addition on the differential bacterial microbiota under different nitrogen levels. At the phylum level, compared with treatment N0, BN0 significantly increased the relative abundances of *Actinobacteriota*, *Bacteroidota*, and *Nitrospira* (Figure 6a). Similarly, compared with treatment N100, BN100 significantly increased the relative abundances of *Chloroflexi*, *Bacteroidota*, and *Nitrospira* (Figure 6b). At the genus level, compared with treatment N0, BN0 significantly increased the relative abundances of *Bacillus*, *Gemmatimonas*, *Streptomyces*, and *Nitrospira* (Figure 6c). Compared with treatment N100, BN100 significantly increased the relative abundances of *Sphingomonas*, *Bacillus*, *Gemmatimonas*, and *Paenibacillus* (Figure 6d). The results indicated that biochar addition altered the soil bacterial community composition and increased the relative abundances of nitrogen metabolism-related microbes such as *Nitrospira*, *Sphingomonas*, and *Paenibacillus*, thereby influencing soil nitrogen transformation.

**Figure 7:**
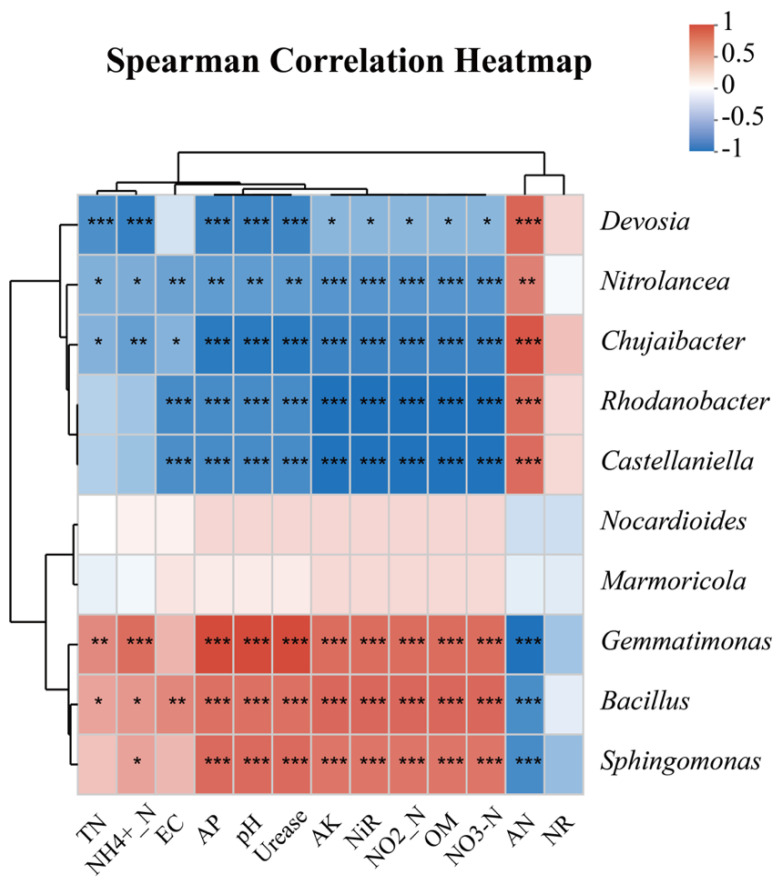
Heat map of correlation between soil chemical properties and bacterial community by biochar addition. Note: * at the 0.05 (two-tailed) level, ** at the 0.01 level (two-tailed), *** *p ≤* 0.001.

Spearman’s correlation analysis was performed to investigate the relationships between the top 10 dominant bacterial genera and soil chemical properties. Soil pH, available phosphorus (AP), available potassium (AK), nitrite nitrogen (NO_2_^−^-N), nitrate nitrogen (NO_3_^−^-N), organic matter (OM) contents, as well as soil urease (UE) and nitrite reductase (NIR) activities, exhibited extremely significant positive correlations with the relative abundances of *Gemmatimonas*, *Bacillus*, and *Sphingomonas* (Figure 7). Soil ammonium nitrogen (NH_4_^+^-N) content showed a significant positive correlation with the relative abundance of *Sphingomonas*. Additionally, soil total nitrogen (TN) and ammonium nitrogen (NH_4_^+^-N) contents were significantly positively correlated with the relative abundances of *Gemmatimonas* and *Bacillus*, but significantly negatively correlated with the relative abundances of *Devosia*, *Nitrolancea*, and *Chujaibacter*.

## 3. Discussion

Excessive soil nitrogen inhibits normal plant growth [22,23,24]. In this study, over-application of nitrogen decreased cucumber plant height, reduced chlorophyll content, and restricted root growth. In contrast, treatment BN100 increased cucumber root dry weight, total root length, root surface area, and root volume. It also enhanced leaf chlorophyll content and promoted overall plant growth. This may be because biochar immobilizes soil nitrogen through its porous structure, promotes nitrogen transformation [25] and improved the root growth environment. Furthermore, biochar also optimizes the rhizosphere microbial ecological environment, enriches beneficial microorganisms [26], enhance the root system’s ability to absorb nutrients, stimulate the synthesis of chlorophyll in leaves [27,28], increase the energy required for plant metabolism, and ultimately promote the growth of cucumber plants [29].

Plant root nitrogen absorption capacity depends both soil nitrogen content and internal root nitrogen metabolism [30,31]. In the present study, the BN100 treatment increased nitrate nitrogen content while decreasing ammonium nitrogen content in cucumber roots. This may be because excessive nitrogen leads to soil nitrogen accumulation and delays root nitrate nitrogen assimilation and reduction, thus increasing plant nitrate nitrogen levels [32]. Additionally, previous studies have demonstrated that exogenous carbon application enhances the activities of nitrate reductase (NR) and GOGAT in roots, which promotes nitrate nitrogen uptake and strengthens ammonium nitrogen assimilation capacity, consequently reducing root ammonium nitrogen content [33,34]. The increased NR and GOGAT activities in BN100 further confirm that biochar promotes root ammonium nitrogen assimilation and conversion to nitrate nitrogen, improving root nitrogen uptake and transformation efficiency.

A favorable soil environment is a prerequisite for crop growth and development. However, long-term nitrogen application degraded soil quality and caused nitrogen loss [35]. Biochar improves the soil environment by increasing soil pH and organic matter content, thereby regulating soil nitrogen transformation processes [36]. In this study, BN100 reduced soil ammonium nitrogen and increased nitrate and nitrite nitrogen contents. Biochar acts as an exogenous carbon source, which optimizes the microbial ecological environment, promotes the propagation and activity of beneficial soil bacteria [36,37], enhance the activities of soil nitrogen-metabolizing enzyme activities [38], thereby modulating soil nitrogen transformation. Furthermore, this study found that the BN100 treatment enhanced soil nitrogen-metabolizing enzyme activities, indicating that this treatment promotes nitrogen transformation through multiple pathways: urease hydrolyzes nitrogen to produce ammonia, NPA accelerates nitrogen mineralization to facilitate the conversion of organic nitrogen to inorganic nitrogen; meanwhile, nitrite reductase and nitrate reductase drive the conversion of soil ammonium nitrogen to nitrite nitrogen and nitrate nitrogen, ultimately increasing soil nitrite nitrogen and nitrate nitrogen contents [39,40].

Biochar application significantly increased soil pH, which played a key regulatory role. The increased pH alleviated soil acidification caused by excessive nitrogen, optimized the microenvironment for enzyme catalysis and microbial survival, and enhanced the activities of nitrogen metabolism enzymes. The pH increase also promoted the growth of *Nitrospira*, *Sphingomonas*, *Bacillus* and other beneficial bacteria, forming a positive feedback loop of “pH increase → enzyme activity enhancement → microbial optimization → nitrogen transformation improvement” [41]. In this study, the addition of biochar under the N100 treatment increased soil nitrate nitroge content and the relative abundance of *Nitrospirota*, which is inconsistent with the findings of Sun et al. [42]. This may be attributed to the adsorption capacity of biochar, which enhances the uptake of soil nutrients by bacterial microorganisms, and the increase in microbial activity is associated with nitrate nitrogen content [43,44]. Furthermore, the elevated soil nitrate nitrogen content following biochar application provides more nutrients required for nitrifying microbial activity, thereby increasing the relative abundance of *Nitrospirota*. This differs from some reports that biochar reduces nitrifier abundance; the discrepancy may stem from soil acidity, N rate, and biochar type. Our results confirm that biochar interacts with optimal N (100 kg·hm^−2^) to selectively boost N-cycling microbes in acidic facility soil.

Microbial community changes are closely related to variations in the soil environment, and alterations in soil chemical properties are important factors influencing the structure and composition of bacterial communities [45,46]. Increasing soil nitrogen content typically reduces bacterial community abundance and diversity, whereas biochar application significantly enhanced these parameters. Furthermore, the community structures of the N0 and N100 treatments were similar but distinctly different from that of the BN0 treatment from the PCA analysis. This indicated that biochar application significantly improved soil bacterial community diversity and abundance. PCA analysis had a limitation: The low total variance explained (22.43%) suggests other unmeasured factors (e.g., fungal communities, soil physical properties) also shape microbial structure, which should be explored in future research. By modifying the bacterial community structure in the cucumber rhizosphere, biochar enhanced soil nutrient content and ameliorates the soil micro-ecological environment, thereby providing a suitable habitat and nutrients for the growth of microbial [47,48]. Consistent with these findings, the correlation heatmap revealed that soil nutrient contents (available phosphorus, available K, NO_2_^−^-N, NO_3_^−^-N), soil pH, and the activities of soil nitrogen-metabolizing enzymes (urease and nitrite reductase) were significantly positively correlated with the relative abundances of *Gemmatimonas*, *Bacillus*, and *Sphingomonas*, which further validated the aforementioned conclusions. Correlations between soil properties and microbes reflect associative patterns rather than causal links. Future studies should use metatranscriptomics or stable isotope probing to verify direct microbial functional impacts on N transformation.

Although BN100 showed the most beneficial effects, the responses of BN150 and BN200 also deserve attention. Compared with N150, BN150 significantly increased soil pH, OM, and AK, and enhanced UE and NPA activities, indicating biochar alleviated the adverse effects of conventional N rates. However, BN150 did not further increase root N metabolism enzyme activities or cucumber growth compared with BN100, likely due to excessive N limiting biochar’s promotional effects. For BN200, although biochar improved soil properties and microbial diversity, the extremely high N rate (200 kg·hm^−2^) still caused soil acidification and reduced NUE, resulting in no significant growth improvement compared with N200. These results suggest that biochar cannot completely offset the negative impacts of excessive N, and an appropriate N rate (e.g., 100 kg·hm^−2^) is a prerequisite for maximizing biochar’s benefits in facility cucumber production.

## 4. Materials and Methods

### 4.1. Materials

The experiment soil had not been planted with cucurbitaceae crops previously. The soil had a pH of 5.76, EC of 132.83 µS·cm^−1^, organic matter content of 29.87 g·kg^−1^, alkali-hydrolyzable nitrogen content of 79.11 mg·kg^−1^, available potassium (K) content of 181.00 mg·kg^−1^, and available phosphorus (P) content of 121.08 mg·kg^−1^. The cucumber seeds ‘Tianjiao 8’ was provided by Shuofengyuan Seed Co., Ltd., Qingdao, China.

The experiment biochar was produced by pyrolyzing corn stover under oxygen-limited conditions at 350–550 °C (Liaoning Provincial Biochar Engineering Technology Center, Shenyang, China). Its basic physicochemical properties were as follows: average pore size 16.27 nm, particle size 1.5–2.0 mm, total carbon content 703 g·kg^−1^, total N content 15.3 g·kg^−1^, and pH 8.9. The urea fertilizer (46% N), and compound fertilizer (N-P_2_O_5_-K_2_O = 15-15-15) provided by Xindu Chemical Compound Fertilizer Co., Ltd., Yingcheng, China. It should be noted that 5% (*w*/*w*) biochar is approximately 100 t·ha^−1^ at field scale, which is much higher than conventional field application rates (1–10 t·ha^−1^). Therefore, the results of this study are more suitable for mechanistic revelation, and field application should be further adjusted according to actual soil conditions.

### 4.2. Experimental Design

The experiment was conducted from June to September 2024 at the Horticulture and Landscape Experimental Station of Hebei Normal University of Science and Technology (39°42′ N, 119°10′ E, altitude 22 m). The experiment consisted of eight treatments with three biological replicates arranged in a randomized complete block design. The urea was used as the nitrogen fertilizer and four levels were set up to simulate soils with different nitrogen contents: N0 (no nitrogen fertilizer), N100 (nitrogen fertilizer with 100 kg·hm^−2^), N150 (nitrogen fertilizer with 150 kg·hm^−2^), and N200 (nitrogen fertilizer with 200 kg·hm^−2^), with the addition of 5% biochar (BN0, BN100, BN150, BN200) (Table 3). P and K fertilizer levels were consistent across all treatments. The N application rates (100, 150, and 200 kg N ha^−1^) were selected based on local cucumber fertilization recommendations (120–180 kg N ha^−1^ for optimal yield), previous studies on excessive N accumulation in greenhouse soils, and preliminary pot trials. Specifically, 100 kg N ha^−1^ represented a moderate rate, 150 kg N ha^−1^ a conventional rate, and 200 kg N ha^−1^ an excessive rate commonly observed in farmers’ fields.

Each treatment contained three biological replicates, and each replicate included six pots; one cucumber seedling was planted per pot. Seedling raising began on 6 June 2024, with a seedling period of 28 days. When the cucumber seedlings had two leaves with one heart, urea mixed with 5% biochar (by mass) and 10 g of compound K sulfate fertilizer were evenly mixed and added to the planting pots (top diameter 26.6 cm, bottom diameter 21.2 cm, height 24.5 cm with 600 g of sieved soil) in one application, and one plant per pot. The same amount of water was supplied during the 45-day cucumber growth period (4 July to 18 August 2024). Other management practices were consistent with local conventional production. All fertilizers were applied once at transplanting to simulate the common practice of base fertilization in intensive facility cucumber production, avoiding the confounding effects of split N application on soil N dynamics.

### 4.3. Determination Indexes and Methods

Forty-five days after cucumber planting, three plants with consistent growth were randomly selected from each treatment to measure the height, stem diameter, and leaf chlorophyll content. Cucumber plants were sampled 45 days after transplanting (vegetative to early flowering stage), a critical period for root growth and nutrient uptake. The roots of the plant were washed clean by ultrapure water and then mixed and divided into two portions. One portion was used for the determination of root dry weight and nitrogen content, the other portion was stored at −80 °C freezer for subsequent determination of nitrogen metabolism enzyme activity. After removing the roots and impurities, the soil was mixed and sieved (40-mesh screen) into three portions. One portion was stored in a 4 °C refrigerator for the determination of soil ammonium nitrogen, nitrate nitrogen, and nitrite nitrogen content. Another portion was air-dried indoors and sieved (40-mesh screen) for the determination of soil chemical properties and soil enzyme activity. Five portions were divided using sterile centrifuge tubes, quickly frozen by liquid nitrogen, and stored in a −80 °C ultra-low temperature freezer for microbial diversity sequencing.

Chlorophyll content was measured using a SPAD-502 portable chlorophyll meter (Konica Minolta, Tokyo, Japan). Three fully expanded functional leaves were selected from each plant, and five points were measured on each leaf; the average value was taken as the SPAD value representing chlorophyll content.

Soil pH and EC were measured by mixing soil with deionized water at 1:5 (*w*/*v*) with a multi-parameter water quality analyzer (DZS-708, Leici Co., Ltd., Shanghai, China). Soil organic matter content was determined using the K dichromate-volumetric dilution method [49]. Total nitrogen content was determined by an elemental analyzer (Vario EL elemental analyzer, Hanau, Germany) [50]. Root nitrate nitrogen and ammonium nitrogen content were determined by a continuous flow analyzer (BRAN + LUEBBE AutoAnalyzer3, Hamburg, Germany) [51]. Available P, available K content was determined by the method of Bao [52], Available P was determined by the Olsen method (0.5 mol L^−1^ NaHCO_3_ extraction). Available K was determined by the ammonium acetate extraction method (1 mol L^−1^ NH_4_OAc, pH 7.0). Nitrate reductase (NR) and GOGAT enzyme activity of the root were determined using a kit (Solarbio Science & Technology Co., Ltd., Beijing, China). Soil enzyme activities (Urease, Neutral protease, Nitrate reductase, Nitrite reductase) were determined according to soil enzyme research methods [53].

Soil microbial diversity sequencing was entrusted to Majorbio Bio-Pharm Technology Co., Ltd. (Shanghai, China). Mothur (v1.30.2) was used for microbial alpha diversity analysis. PCA analysis was performed to show differences in microbial community structure. The Wilcoxon rank-sum test was used to analyze microbial differences. Spearman correlation coefficients between environmental factors and microorganisms were calculated, and heatmaps were generated using R (v3.3.1).

### 4.4. Statistical Analysis

Data were analyzed using Microsoft Excel 2019 and GraphPad Prism 9. One-way analysis of variance (ANOVA) was performed for plant growth parameters, soil chemical properties, N fractions, enzyme activities, and microbial diversity indices. Significant differences among treatments were identified using Duncan’s multiple range test (*p* < 0.05). All figures were generated using GraphPad Prism 9 and Origin 2022.

## 5. Conclusions

In conclusion, biochar application effectively improved soil nitrogen transformation and promoted cucumber growth under facility cultivation conditions. The optimal treatment was 100 kg·hm^−2^ nitrogen combined with 5% biochar (BN100). Biochar increased soil pH, alleviated soil acidification, and enhanced the activities of nitrogen-metabolizing enzymes in the soil, thereby increasing nitrate and nitrite nitrogen contents. It also enhanced the activities of key enzymes involved in root nitrogen metabolism, improved root nitrogen metabolic capacity, and promoted plant growth. Furthermore, biochar application with N100 improved the ecological environment of soil bacteria, increased the relative abundances of nitrogen-metabolizing microorganisms such as *Nitrospirota*, *Nitrospira*, and *Sphingomonas*, and facilitated nitrification and nitrogen fixation processes in the soil. The results provide a scientific basis for rational nitrogen fertilizer application and biochar utilization in facility cucumber production.

## Figures and Tables

**Table 1:** Effects of biochar–nitrogen interaction on soil chemical properties.

Treatment	pH	Organic Matter Content (g·kg^−1^)	Alkaline N Decomposition Content (mg·kg^−1^)	Available P Content (mg·kg^−1^)	Available K Content (mg·kg^−1^)
N0	5.27 ± 0.03 d	26.34 ± 0.50 c	76.14 ± 4.21 d	138.39 ± 4.82 bc	270.00 ± 1.63 e
N100	5.19 ± 0.02 e	26.98 ± 0.81 c	85.66 ± 6.57 d	137.08 ± 2.26 bc	213.00 ± 1.63 h
N150	5.15 ± 0.02 e	26.44 ± 0.82 c	116.60 ± 1.46 b	134.84 ± 4.85 c	238.00 ± 4.32 g
N200	4.99 ± 0.05 f	25.52 ± 1.09 c	127.84 ± 0.44 a	165.62 ± 2.43 a	255.33 ± 1.70 f
BN0	5.67 ± 0.04 b	41.70 ± 0.25 ab	76.73 ± 3.37 d	146.23 ± 2.15 b	446.50 ± 3.67 b
BN100	6.01 ± 0.01 a	41.36 ± 0.41 b	76.14 ± 0.84 d	146.07 ± 3.35 b	330.33 ± 0.47 d
BN150	5.28 ± 0.02 d	40.88 ± 0.81 b	91.61 ± 4.37 d	140.51 ± 3.95 bc	488.00 ± 2.94 a
BN200	5.51 ± 0.02 c	42.99 ± 0.25 a	97.56 ± 3.67 c	136.39 ± 5.94 bc	386.25 ± 3.47 c

Note: Different lowercase letters after the data in the same column indicate significant differences between treatments (*p* < 0.05). The following table is the same.

**Table 2:** Effects of biochar–nitrogen interaction on soil nitrogen fractions.

Treatment	Total Nitrogen Content (mg·kg^−1^)	Ammonium Nitrogen Content (mg·kg^−1^)	Nitrite Nitrogen Content (μmol·g^−1^)	Nitrate Nitrogen Content (mg·kg^−1^)
N0	1.52 ± 0.03 d	8.38 ± 1.56 e	1.77 ± 0.09 e	342.81 ± 7.22 a
N100	1.70 ± 0.01 c	10.85 ± 2.55 e	3.08 ± 0.08 b	343.71 ± 9.15 a
N150	1.87 ± 0.06 b	63.28 ± 2.87 b	3.12 ± 0.06 b	324.08 ± 8.86 b
N200	1.72 ± 0.06 c	26.95 ± 4.86 d	2.63 ± 0.30 c	294.81 ± 14.34 c
BN0	1.86 ± 0.05 b	11.83 ± 1.60 d	1.99 ± 0.10 e	351.18 ± 3.33 a
BN100	1.85 ± 0.01 b	5.97 ± 0.44 e	3.58 ± 0.04 a	356.04 ± 7.63 a
BN150	1.94 ± 0.03 ab	43.68 ± 0.20 c	2.93 ± 0.09 b	353.39 ± 4.41 a
BN200	2.00 ± 0.02 a	72.35 ± 4.28 a	2.31 ± 0.16 d	348.13 ± 6.67 a

Note: Different lowercase letters after the data in the same column indicate significant differences between treatments (*p* < 0.05).

**Table 3:** Simulation of soil values for different nitrogen treatments.

Treatment	Biochar %	Nitrogen Fertilizer Input (kg·hm^−2^)
N0	0	0
N100	0	100
N150	0	150
N200	0	200
BN0	5	0
BN100	5	100
BN150	5	150
BN200	5	200

## Data Availability

Data is contained within the article and Supplementary Materials.

## Supplementary Materials

The following supporting information can be downloaded at: https://www.mdpi.com/article/10.3390/plants15111658/s1, Table S1. Effect of biochar added on the plant growth of cucumber; Table S2. Soil microbial sequencing data statistics; Figure S1. Effect of biochar added on the bacteria community with soils impact of Alpha diversity.
