# Supplementary material for: Effects of Biochar–Nitrogen Interaction on Soil Nitrogen Transformation and Cucumber Growth in Facility Cultivation
Source: Plants (Basel). 2026 May 28;15(11):1658. doi: 10.3390/plants15111658 (PMC13259239; doi:10.3390/plants15111658)
Supplement: Supplementary file 1 [file plants-15-01658-s001.zip › plants-4260052-supplementary.pdf]

Table S1. Effect of biochar added on the plant growth of cucumber

| Treatment | Plant height (cm) | Stem diameter (mm) | chlorophyll content (SPAD) | shoot dry weight (g) | root dry weight (g) |
|---|---|---|---|---|---|
| N0 | 176.13±3.42c | 9.05±0.68abc | 56.93±3.73b | 16.81±1.12ab | 0.58±0.008ab |
| N100 | 190.90±5.16b | 9.13±0.77ab | 53.42±4.60bc | 20.75±1.06a | 0.51±0.09ab |
| N150 | 190.68±8.46b | 7.93±0.21c | 109.91±0.64a | 15.05±1.10bc | 0.42±0.07ab |
| N200 | 177.32±6.14c | 8.17±0.74bc | 106.27±3.10a | 13.99±0.23bc | 0.37±0.16b |
| BN0 | 213.18±9.80a | 9.48±0.93a | 48.80±2.93c | 16.72±2.14ab | 0.58±0.08ab |
| BN100 | 221.03±8.60a | 8.54±0.73abc | 111.47±5.68a | 18.02±2.72ab | 0.69±0.13a |
| BN150 | 172.58±9.01c | 8.96±0.70abc | 106.37±0.69a | 11.47±0.26c | 0.34±0.16b |
| BN200 | 174.62±5.99c | 9.04±0.85abc | 104.92±3.63a | 13.96±0.14bc | 0.44±0.06ab |

Note: Different lowercase letters after the data in the same column indicate significant differences between treatments ($P<0.05$). The following table is the same.

Table S2. Soil microbial sequencing data statistics

| Sample | Number of optimized sequences | Number of optimized bases | Mean length/bp | Minimum length/bp | Maximum length/bp |
|---|---|---|---|---|---|
| N0_1 | 64846 | 26936862 | 415.397434 | 257 | 511 |
| N0_2 | 63172 | 26255247 | 415.615257 | 345 | 524 |
| N0_3 | 62276 | 25903728 | 415.950414 | 215 | 455 |
| N0_4 | 65393 | 27181037 | 415.656676 | 252 | 512 |
| N0_5 | 66711 | 27713799 | 415.430724 | 297 | 504 |
| N100_1 | 62493 | 26040902 | 416.701103 | 222 | 526 |
| N100_2 | 63311 | 26385298 | 416.75693 | 247 | 475 |
| N100_3 | 62985 | 26275288 | 417.167389 | 219 | 468 |
| N100_4 | 61606 | 25650416 | 416.362302 | 277 | 473 |
| N100_5 | 61713 | 25719130 | 416.753844 | 282 | 512 |
| BN0_1 | 70893 | 29516997 | 416.359824 | 213 | 494 |
| BN0_2 | 75301 | 31310576 | 415.80558 | 203 | 466 |
| BN0_3 | 70596 | 29349145 | 415.733823 | 232 | 455 |
| BN0_4 | 63139 | 26259361 | 415.897639 | 291 | 491 |
| BN0_5 | 57239 | 23842225 | 416.538112 | 203 | 511 |
| BN100_1 | 68938 | 28678768 | 416.008123 | 235 | 453 |
| BN100_2 | 71444 | 29711119 | 415.865839 | 245 | 528 |
| BN100_3 | 66045 | 27438009 | 415.444152 | 327 | 452 |
| BN100_4 | 68953 | 28669478 | 415.782896 | 212 | 525 |
| BN100_5 | 66057 | 27450745 | 415.561485 | 341 | 509 |

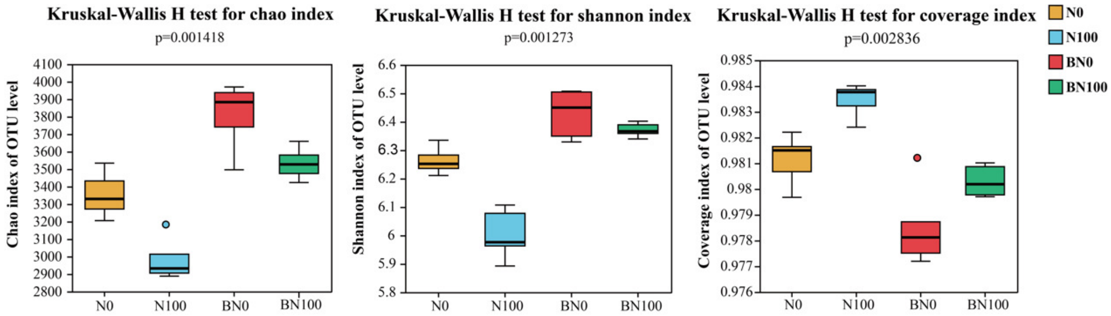

Figure S1. Effect of biochar added on the bacteria community with soils impact of Alpha diversity
